# Weathering the rural reality: delivery of the Nurse-Family Partnership home visitation program in rural British Columbia, Canada

**DOI:** 10.1186/s12912-019-0341-3

**Published:** 2019-05-02

**Authors:** Karen A. Campbell, Karen MacKinnon, Maureen Dobbins, Natasha Van Borek, Susan M. Jack, For the British Columbia Healthy Connections Project Process Evaluation Research Team, Nicole Catherine, Andrea Gonzalez, Christine Kurtz Landy, Harriet MacMillan, Lenora Marcellus, Debbie Sheehan, Lil Tonmyr, Colleen Varcoe, Charlotte Waddell

**Affiliations:** 1 School of Nursing, McMaster University, Hamilton, Ontario Canada; 2 School of Nursing, University of Victoria, Victoria, British Columbia Canada

**Keywords:** Public health nursing, Rural, Nurse-family partnership, Interpretive description, Home visitation

## Abstract

**Background:**

Pregnant girls/young women and new mothers living in situations of social and economic disadvantage are at increased risk for poor health. Rural living may compound marginalization and create additional challenges for young mothers. Public health nurses (PHNs) delivering the Nurse-Family Partnership (NFP) to mothers living in rural communities may help to improve maternal and child health outcomes. The purpose of this analysis, grounded in data collected as part of a broader process evaluation, was to explore and understand the influence of rural geography on the delivery of NFP in British Columbia, Canada.

**Methods:**

For the analysis of this qualitative data, principles of inductive reasoning based on the methodology of interpretive description were applied. A total of 10 PHNs and 11 supervisors providing the NFP program in rural communities were interviewed.

**Results:**

The results of this analysis reflect the factors and challenges of providing the NFP program in rural communities. PHNs noted the importance of NFP in the lives of their rural clients, especially in the face of extreme financial and social disparity. Remaining flexible in their approach to rural nursing and protecting time to complete NFP work supported nurses practicing in rural environments. Rural PHNs were often the sole NFP nurse in their office and struggled to remain connected to their supervisors and other NFP colleagues. Challenges were compounded by the realities of rural geography, such as poor weather, reduced accessibility, and long travel distances; however, these were considered normal occurrences of rural practice by nurses.

**Conclusions:**

PHNs and NFP supervisors are well-positioned to identify the modifications that are required to support the delivery of NFP in rural geography. NFP nurses need to articulate what classifies as rural in order to effectively determine how to best provide services to these populations. Environmental conditions must be considered when offering NFP in rural communities, particularly if they impact the time required to deliver the program and additional services offered to young mothers. Regular NFP meetings and education opportunities address common problems associated with rural nursing but could be enhanced by better use of technology.

**Electronic supplementary material:**

The online version of this article (10.1186/s12912-019-0341-3) contains supplementary material, which is available to authorized users.

## Background

Mothers of young maternal age who are living in poverty and/or social deprivation are at increased risk for poor health outcomes across the lifespan of the mother and her children [1]. Nurse-Family Partnership® (NFP) is an early intervention program shown to improve child and maternal health through nurse home visiting with young, first-time mothers experiencing social and economic disadvantage [2]. This population is considered to be particularly vulnerable when they have also experienced poverty in childhood, low education attainment, underemployment, and violence across the lifespan [1]. Given the widening gap in health and social inequalities that occurs when there is a perpetuation of disadvantage, it is imperative that supports are made available to this population of young mothers [1]. Rural living may potentially compound marginalization and create additional challenges for young mothers and their children. Rural residents have poorer health status, fewer available health resources, and greater difficulty accessing health services despite their significant need for primary health care [3–6].

In Canada, the provision of home visiting services by public health nurses (PHNs) is a strategy used to enhance access to health promotion and injury prevention services, particularly to improve reproductive and child health outcomes [7]. NFP is a specific nurse home visitation program that was developed and extensively evaluated in the United States. The NFP intervention starts early in pregnancy with intensive and purposeful home visits that continue until the child’s second birthday. There are specific program elements outlined for nurses, supervisors, and organizations involved in implementation of NFP. This includes guidance and requirements for client enrollment criteria, intervention delivery, home visit content, nurse/supervisor education, supervision and team activities (See Table 1) [8, 9]. An extensive process evaluation is currently being conducted in British Columbia, Canada to document how NFP is implemented and delivered within this context [8]. The process evaluation is adjunctive to the British Columbia Healthy Connections Project (BCHCP), the first Canadian randomized controlled trial (RCT) evaluating the effectiveness of NFP [10]. If the intervention is shown effective, the findings from the process evaluation will be used to inform adaptations necessary to ensure that this program meets the needs of Canadian mothers, reflects PHN competencies, and is feasible to deliver across a range of geographic contexts [8, 11]. The purpose of this analysis, grounded in data collected as part of the broader process evaluation, was to explore and understand the influence of rural geography on the delivery of the NFP program in British Columbia, Canada.

**Table 1:** NFP Program Model Elements for British Columbia

Interventionist	• PHNs and nurse supervisors are Registered Nurses with a minimum of a baccalaureate degree in Nursing
	• PHNs and nurse supervisors complete educational sessions to develop core NFP competencies, and participate in ongoing learning activities
	• NFP PHNs use professional judgement, skill, and knowledge to individualize care based on family strengths and risks and across six domains of the program
	• Nurse supervisors provide clinical supervision with regular (weekly) reflection, demonstrate integration of the theories, and facilitate professional development essential to the PHN home visitor role
	• Specific supervisory activities include one-to-one clinical supervision, case conferences, team meetings, and field supervision
Client eligibility	• Clients participate voluntarily, are a first-time mother, meet socio-economic disadvantage criteria at intake, is enrolled no later than week 28 of pregnancy
	• Clients are 24 years of age or younger at time of enrollment
Dose	• Client is visited one-to-one, one PHN to one first-time mother or family
	• Client is visited in her home or occasionally in another setting that is mutually determined between the PHN and the client
	• Full-time PHNs have no more than 20 active clients
	• A full-time supervisor is responsible for a team with a maximum of 8 NFP PHNs
Visit Schedule	• General guidance is provided about a visit schedule (see below); however, there is flexibility to alter the schedule to meet maternal needs, availability, and priorities.
	• Upon enrollment, four weekly visits then bi-weekly until delivery
	• Post-partum, six weekly visits then bi-weekly until infant is 21 months
	• Monthly visits from 21 to 24 months
Program Domains (Home visit content)	• Within each home visit, a PHN will review and discuss content from six domains: 1) personal health; 2) environmental health; 3) life course development; 4) maternal role; 5) friends and family; and, 6) health and human services

Adapted from Jack et al. [8] and Prevention Research Center for Family and Child Health [9]

While there are few studies that have focused on the practices of rural PHNs in Canada and the United States, those that are available provide some insight into the nature of nurses’ experiences. Rural PHNs often practice as generalists and are cross-trained to provide a wide array of services [12, 13]. When providing maternity services, rural PHNs conduct newborn, maternal, and family assessments, provide breastfeeding support, and answer questions related to maternal health and infant care [13].

Rural PHNs are strategic in their client and community relations, allowing themselves to be known in the community to build and establish trust within the community [14, 15]. PHNs tend to take on leadership roles in community development and form strong collaborative relationships with community members and organizations to ensure that rural clients’ needs are met [13, 16]. Rural nurses often feel a responsibility to their communities to ensure that healthcare services are available and accessible [13, 15, 17]. Where rural nurses are also a community member, strong ties to their neighbourhoods can create complicated relationships with clients who they may encounter outside of work or have additional knowledge of the family beyond what is shared in their nursing assessment [13, 15, 17, 18]. The lack of anonymity associated with living in rural communities is a concern for young mothers who may be hesitant to reveal their true situations for fear of breached confidentiality and rural nurses need to be mindful of not inadvertently exposing information [19].

Home visiting is an evidence-based public health strategy that has been shown to increase maternal well-being, improve child health outcomes, and reduce child maltreatment among mothers experiencing social and economic disadvantage [2, 20–26]. Most notably in the United States, evaluations of NFP, conducted across three RCTs, demonstrated consistent and enduring effects related to immediate and long-term health and well-being outcomes for mothers and their children [2]. For mothers participating in NFP, immediate benefits included: 1) positive changes in prenatal behaviours, such as improved diet; 2) lowered use of cigarettes; 3) increased formal and informal support networks; and 4) improved breastfeeding initiation [2]. Child benefits included: 1) an increase in infant birth weight; 2) reduction of child injuries and ingestions that may have been associated with child abuse and neglect; and, 3) improved emotional, cognitive, and language development [2, 24, 27, 28]. The long-term benefits for mothers and infants are also documented as fewer sexual partners, fewer arrests, and lower violations of probation for adolescent children of NFP-visited mothers; improved life-course of mothers, specifically related to reduction and spacing of subsequent pregnancies, less role impairment due to drugs or alcohol, increased participation in the work-force, experiencing less domestic violence, and not being dependent on social welfare; and reduced all-cause mortality in mothers and preventable-cause mortality in children [2, 24, 27, 28].

Trials of NFP conducted outside of the United States showed varied outcomes [29, 30]. Results from the Netherlands indicated many positive health and social outcomes for new mothers and their infants, including reduction in prenatal smoking, increased breastfeeding, reduced child protection reports, and reduced exposure to intimate-partner violence [31–33]. In contrast, findings from the England trial indicated no additional benefits to mothers in NFP [34]. NFP trials are a prerequisite for any international expansion of the program because context is essential for guiding policy and practice [35].

While trials in both the Netherlands and England included rural areas neither reported the influence of geography on NFP delivery or outcomes. With the exception of the initial NFP trial in Elmira, NY, evaluations in the United States largely occurred in urban settings. Only one study focused on the implementation of NFP home visiting in rural communities [36]. Rubin and colleagues conducted a large retrospective cohort study with 3844 NFP clients (urban *n* = 3296; rural *n* = 548) matched to 10,938 control subjects to determine the influence of NFP on the reduction of subsequent pregnancies across different geographical locations. While the findings revealed a reduction in second pregnancy rates among all NFP clients, they were twice as strong in younger mothers from rural locations (hazard ratio = 0.40; 95% confidence interval, 0.22–0.73). Though this study provides important information into the effectiveness of NFP in rural communities, examination of the practices of NFP nurses could provide a better understanding of how the program is adapted or modified in rural settings. Given the importance of context, there is added value in attending to geographical considerations.

Understanding how rural PHNs adapt, implement, and deliver home-visiting to clients in rural communities is necessary for program success, but limited evidence exists to support rural nursing practice. One study suggested that generic training provided to PHNs for home-visiting programs may not adequately address the demands or needs of families living in rural communities [37]. From an organisational perspective, rural nurses have greater difficulty receiving training opportunities and lack consistent supervision, thereby missing opportunities for the shared-decision making that may occur in metropolitan centres [37]. Addressing rural nursing issues and concerns may help contextualize home-visiting programs and promote geographically-appropriate interventions for rural communities.

The literature reviewed here has provided an overview of the evidence that exists to support and inform the practices of PHNs working in rural communities. Rural nurses have relationships within their communities and have a commitment to maintaining health services in their rural communities. However, little is known about how nurses’ practices are influenced by their geographical location.

## Methods

### Rural definitions

The body of literature defining the term rural is broad and there is no consensus on a standard definition. Rural has been defined by researchers using a variety of classifications, including community characteristics, geographical location, and availability or accessibility of health and technological resources [38]. For the purposes of this study, the rural context is being conceptualized as a continuum that encompasses a wide variety of geographic factors that range from small towns to farmland to isolated communities and/or any cultural variables. Using a broad definition allowed for participants’ voices to guide the conceptualization of rural geography used for the analysis that follows.

### Ethics

The BCHCP process evaluation received research ethics board approvals from 10 institutions, including: five participating regional British Columbia health authorities; four universities where researchers held their faculty appointments or affiliations; and the Public Health Agency of Canada. All study participants provided informed consent to the research coordinator (NV) who explained the study objectives and their involvement in the study was voluntary. Both written and verbal consent was obtained. The nurses’ consent form included information about their role in delivering the NFP program that was not found on the supervisor consent; otherwise, all information was consistent for all participant consent forms. Participants were advised that reflecting on experiences during the interview may cause emotional distress, and as such, were given the opportunity to take a break or stop interviews at any time. All data remained confidential, with identifying information removed from transcripts and attention to ensuring that individual nurses and supervisors could not be identified in any outcome reports.

### Design

For the analysis of this qualitative study, principles of inductive reasoning based on the methodology of interpretive description were applied [39]. This approach was particularly appropriate for this study because its purpose is to generate knowledge to improve clinicians’ understanding of healthcare challenges and provide clinically relevant applications to practice [39, 40]. As an applied qualitative approach, interpretive description draws on the knowledge generated from the discipline engaging in the research project [39]. In this study, the epistemology of nursing served as the theoretical scaffolding rather than arbitrarily applying a framework ill-suited to understand the specific disciplinary knowledge and complex context of rural PHNs in the NFP program [39]. This method requires that the researcher designs a study for the purpose of answering a clinically-relevant research question and identifies a sample that is best able to identify an array of insights into a complex phenomenon [39].

### Sample

Sampling in interpretive description can include a variety of approaches including purposive and theoretical [39]. For this process evaluation purposive sampling was used to identify participants who were capable of providing deep insight into the phenomenon of rural nursing within NFP delivery. NFP was implemented in five regional health authorities across one province, British Columbia, and delivered by PHNs employed at the health authority and assigned to deliver NFP. NFP core model elements require that each NFP PHN has a supervisor specific to NFP, with a ratio of no more than eight PHNs allocated to one NFP supervisor [11]. For the process evaluation, data were collected from PHNs delivering the NFP program. To provide further insights into the experiences of rural delivery of NFP, the immediate supervisors of NFP PHNs were also invited to participate. All PHNs who were delivering the NFP intervention to participants enrolled in the BCHCP process evaluation were eligible to participate in the study. The total population of supervisors within the NFP program was invited via email to participate in one-to-one interviews.

### Data collection

Data for this analysis were collected during in-depth, semi-structured one-to-one interviews with PHNs who were delivering the NFP to clients enrolled in the BCHCP process evaluation, and NFP supervisors. Participants were interviewed either in person or via telephone, at their workplace by the research coordinator for the BCHCP process evaluation (NV) between October and November of 2014, in the second of eight waves of process evaluation interviews. A demographic questionnaire was completed at the time of the initial interview and field notes were collected by the researcher coordinator (NV). Interviews were an average length of 73 min (range 44–118 min). These interviews had multiple foci; however, for the purpose of this analysis, any content directly relevant to rural delivery of NFP was examined. Additional file 1 summarizes the pertinent questions about rural practice explored in this wave of interviews. Each participant was provided with interview questions prior to the meeting. However, these questions served only as a guide and participants were free to answer in any way that was meaningful to them. This approach to data collection is congruent with the method of interpretive description, which aims to develop a comprehensive understanding of the phenomenon under investigation [39]. Interviews were audio recorded, transcribed verbatim with all identifying information removed by a professional transcriptionist, and not shared with the participants.

### Data analysis

Data analysis in the interpretive description tradition involves deep engagement with the data and encourages the employment of multiple strategies to test, confirm, explore, and expand on developing theories and conceptualizations, as well as to develop practical clinical applications [39]. Interpretive description encourages the researcher to begin with what is known, remain open to new ways of understanding the phenomenon, and expand the disciplinary knowledge base related to the topic [39]. An iterative, recursive, and non-linear process of thematic analysis was adopted and is congruent with data analysis in interpretive description [39]. Although qualitative thematic analysis was primarily employed, other analytical techniques included memoing, selective coding, and creating diagrams. All techniques were utilized to encourage conceptual leaps; specifically, data were considered as individual pieces but then moved to patterns, relationships, and finally as a conceptual whole. This type of analysis allowed for the research team to illuminate the challenges and strengths of delivering the NFP in rural communities. Although multiple coders (KC, NV) participated in coding the data, it was through deep engagement with the data whereby the development of themes began to emerge in a way that provided disciplinary relevance, true to data analysis in interpretive description [39]. Themes were identified through multiple readings of each interview until the first author gained a high level of familiarity with the data and insights about the participant’s experiences were determined. To enhance credibility, thematic analysis was reviewed by multiple researchers on the BCHCP team and discussed for congruence (KC, NV, SJ, KM). Data were organized and managed using NVivo Pro 11 and participant quotes were used to bring meaning to the complexity of their practice. Finally, within the interpretive description tradition, the final analysis should be theoretically sound and credible from the viewpoint of those in the discipline, known as the thoughtful clinician test [39]. Therefore, the researchers (KC, KM, SJ) applied their disciplinarily knowledge, vast experience as public health nurses, rural nurses, and as an NFP educator to determine if the findings resonated with nursing experiences. In addition, the BCHCP scientific team included a national NFP coordinator, nurses, and other NFP experts who reviewed and confirmed validity of the results.

## Results

This focused analysis of data from the BCHCP process evaluation included the total population of PHNs practicing primarily outside of urban centres (*n* = 10) and NFP supervisors who were employed to deliver the program at the time of data collection (*n* = 11). All eligible professionals (*n* = 21) consented to participate in semi-structured interviews with a 100% response rate. At the time of their first BCHCP interview, PHNs averaged 17.8 years of nursing experience (range 3–37 years). Supervisors averaged 27.3 years of nursing experience (range 13–37 years).

After describing how the participants defined rural communities, the six interconnected themes that developed from this phase of analysis are presented. These themes include: 1) Being the Sole NFP Nurse; 2) Weathering Realities; 3) Guarding Time; 4) Staying Connected; 5) Remaining Flexible; and, 6) Providing Essential Services.

### Defining Rural: the context for public health nursing work

The participants in this study self-identified their geographical environment as rural. A variety of descriptors were used to classify the geography as a non-urban setting. Many participants considered the limited accessibility to services a factor in determining its place on the rural/urban continuum. Communities that lacked or had limited transportation infrastructure were considered non-urban and referred to as rural. This included locations only accessed by boat or airplane, and those with limited road access, as described by this PHN:

Our community is accessible only by air and water, so we cannot access the rest of our province by road. So, we are surrounded by ocean primarily, and mountains. And so, anybody who wants to leave our community or come into our community has to do that either by air or on a ferry. We're a relatively small community and we're very isolated. (PHN 6)

The geographical structure of the area including farmlands, areas constricted between ocean and mountains, and proximity to large population centres helped to determine rural status. Additionally, some nurses referred to the population of their assigned small town (less than 17,000) as rural and is consistent with Statistics Canada’s [41] definition of a small population centre, which includes populations less than 30,000. Finally, there was recognition that the classification of geography is on a continuum or non-binary. This range of understanding was reflected in participants’ narratives including, “Well we’re rural. We’re not quite as rural as some places” (PHN 3), and “The bigger centres would say we’re rural … when I hear that we’re rural and remote, those words just don’t sit well with me” (PHN 4). In these discussions, participants revealed that communities often ranged from small towns to farmlands, surrounded by outlying areas that nurses considered to be more (or less) rural.

### Being the sole NFP nurse

Nurse participants reflected on the experience of being the sole NFP nurse in their location and recounted the difficulties associated with delivering NFP in rural areas while being isolated. Nurses who supervised isolated rural NFP nurses also noted the challenges that isolation brought to the role. Being the sole NFP provider in rural areas was particularly stressful for nurses who worked part-time as an NFP PHN and where the remainder of their worktime included assignment to a generalist PHN role. Those working as a generalist PHN also continued to provide other public health services to the community, which may have included flu clinics or other public health nursing roles. This dual role required time management and skillfully seeking out supports.

PHNs in rural offices primarily practiced as both an NFP nurse and a public health generalist. When working within this duality, there was a significant need to balance the requirements of both roles by vigilantly allotting time to complete tasks. However, the demands of the generalist PHN role required nurses to participate in other activities, especially during peak times, as one nurse explained:

Oftentimes things that are offered for the NFP nurses are offered at times when for me I am doing the other portion of my job, which means I may not be available to take advantage of those opportunities ... For example, yesterday … there was an NFP education session being offered and it's flu season here and as a public health nurse flu season is all hands-on-deck. (PHN 9)

Other nurses reported being careful to not schedule generalist PHN nursing work on days when NFP meetings were planned. In rural offices, where nurses often balanced multiple roles, attention to time management was a key consideration for work balance.

Connecting with other nurses or supervisors in the NFP program was the most significant communication concern affected by geography. Nurses were discouraged with rurality creating an inability to have in-person contact with their NFP supervisors. Supervisors were geographically in a different office location than NFP nurses, creating challenges and reflected in this quote, “There’s no supervisor [in my office] ... So that has made it challenging from my perspective with always doing everything by telephone” (PHN 9). Nurses continued to meet despite the challenges of not having face-to-face interactions, primarily by telephone.

Nurses who were the only PHN delivering the NFP program in their office struggled with the associated isolation and lack of connection with other NFP peers. Rural nurses needed to have in-person communication and access to nearby colleagues knowledgeable in the NFP program, so they could share experiences. Despite having a few in-person meetings annually, rural nurses lacked connection with other NFP nurses within their health authority and coveted the ability of others who were able to easily connect and communicate with other NFP nurses, as explained by one participant:

I've had a number of clients who have a lot of crises going on right now and just that, sort of ability to debrief with a colleague who understands the work, is doing the frontline work, would be so valuable if they were, you know, on-site with me. (PHN 3)

This sense of isolation was evident in the narratives, as was the need for face-to-face, dialectic communication with other frontline nurses to cope with the complex health and social concerns of clients. This theme of being the sole nurse crossed over into other findings from this analysis and is recognizable within other themes.

### Weathering realities

PHNs delivering the NFP program in rural communities acknowledged the realities associated with rural nursing practice. Place-based realities, such as limited access to services, extreme weather conditions, and long travel times/distance, were normalized in rural communities for the PHNs and NFP supervisors. Furthermore, the combination of these factors added complexity to already difficult situations, such as needing to travel long distances in bad weather. Nurses experienced the rural reality of practicing in communities that had limited access to health and social services. This influenced their ability to refer to other agencies, creating greater client reliance on the nurse. Lack of accessible transportation was also a reality for clients and required nurses to identify strategies to help support rural clients. PHNs made comments, such as, “I just do a lot of driving” (PHN 1) and others referred to commuting as “not a big deal” (PHN 7), reflecting the normalcy of rural commuting.

Weather conditions influenced the delivery of NFP in rural communities because it was often more extreme than in urban centres. As one PHN explained:

Because we work in a town that does get harsher winters than, than the city … where this is part of our job is just the daily life. We have to change our tires, we take those yack-track things [grips placed over shoes for better traction] with us if we need to get out in an icy place or if it's snowy or something. So that's the way that we've always been (PHN 8).

This participant quote reflects the reality of nurses who require vehicles in good repair and other equipment to manage weather-related conditions and was supported by other PHNs in the study. In addition, it highlights how rural PHNs may not consider this an unusual experience because it is a normal occurrence within their geographical setting. Access to weather-related equipment was essential for rural nurses to deliver the NFP program in their communities, particularly when road conditions were challenging.

Traveling from a rural community was done for two specific purposes: attending required NFP team functions (education, team meetings, and case conferences) and providing client visits. Supervisors accepted extra travel time as a necessity for rural nurses to participate in NFP activities because they were held in urban centres. Normalizing lengthy travel time and distance was noted throughout interviews and evidenced by one supervisor who commented, “… some (rural nurses) just have to travel” (Supervisor [SUP] 7). The travel realities for rural nurses became a consideration for how supervisors planned and scheduled the required NFP team meetings. One supervisor commented, “She needs to leave sooner from meetings, so I say the meetings need to end at such a time. And people who live very close to meetings want the meeting to go longer” (SUP 11). Beyond being aware of the travel needs of rural nurses, supervisors were required to attend to any NFP team tensions by acknowledging that meetings would need to end early to accommodate travel. Establishing team meetings that could fit into the rural nurses’ work schedules was one supportive measure taken by supervisors to ensure inclusion of rural nurses on NFP teams while responding to travel realities.

Although traveling to larger cities for NFP education and training sessions was unavoidable and time-intensive, PHNs perceived driving in their communities to be less stressful than urban commuting. PHNs noted the density of the town’s core area where their clients often lived and usually traveled short distances with minimal traffic, reducing the time they spent traveling for NFP client-based work. PHNs reflected on time-consuming travel for distant home visits, “Throwing [far away town] in there eats up my time” (PHN 3). In regions where the geographical boundaries were vast, PHNs reported that they could potentially have a travel time of up to 8 h if they had clients in distant parts of the catchment areas. However, none of the nurses in this study experienced that extreme distance between clients. When caseloads included clients distant from the nurse’s office, arrangements were made to see those clients on the same day. Effective use of travel time was essential for rural delivery of the NFP program.

Mitigating factors, such as organizational policies or financial burdens that place restrictions on travel, were another consideration for supervisors managing rural PHNs. One supervisor commented, “The geography is a huge issue. And in our health authority we’ve had challenges with travel restrictions, and so for the first year that I was in the program we could only meet by telephone” (SUP 4). Although PHNs and supervisors preferred face-to-face team meetings, some organizations had financial restrictions that prevented rural nurses from traveling long distances to attend NFP required events, such as team meetings and education sessions. In other instances, labour laws and collective bargaining agreements were considerations for traveling PHNs because of the extended work day resulting from travel for face-to-face team activities. Supervisors worked with PHNs to identify strategies and solutions that would achieve compliance with existing policies, such as overnight accommodation or attending by telephone.

### Guarding time

The concept of time as it influenced the delivery of NFP in rural communities was a vital consideration for PHNs and supervisors and apparent throughout all of the interviews with PHNs. Guarding time was necessary in order for rural PHNs to meet the requirements of the NFP program and balance their organizational obligations. The NFP supervisors also acknowledged their need to protect PHN time.

Careful allocation of time was important to ensure that rural nurses could meet the demands of their PHN role. Within the NFP role, these nursing tasks included education sessions, case conferences and team meetings, supervision, and home visits. PHNs were frustrated and challenged by the time needed to complete NFP obligations:

I know for awhile there I was kind of getting resentful of our team meetings and my one-on-one supervised meetings, that they had to be weekly. Because all of this just eats into my time and my time just gets to be quite precious when you think about having to prep and chart and plan for the next [home visit] and, and make sure you get back to [the office] to put all your stuff away at the end of the day, and still be finished on time. (PHN 7)

In addition to increased travel time, PHNs were still expected to find time to meet the full range of required elements of the program, including meetings, reflections, charting, among others. Protecting time for rural nurses was one aspect of delivering the NFP that became a significant part of their nursing role.

Supervisors were also cognizant of time constraints for rural NFP PHNs. Understanding the demands on rural PHNs was an important and supportive consideration from NFP supervisors. Some supervisors who were familiar with the demands of rural nurses provided comfort and support to rural PHNs:

She [the supervisor] understands the geographic isolation that we have and the amount of travel time. So, I think her understanding has really helped with the fidelity of NFP because during our reflection she's able to totally talk about those issues and understand what that's like versus me having a manager that lives in a bigger centre that wouldn't understand. (PHN 10)

Many supervisors appeared to be very aware of the time challenges of rural practice, and the difficulty in protecting and supporting PHNs and their use of time, “I really guard her time, but it is a bit of you know a constant push and pull” (SUP 11). For nurses working in a dual NFP and generalist role, there was potential for inconsistency between two supervisors who may not share a common vision for nurses’ use of time. Collaborative supervision requires a clear and consistent organizational direction for rural nurses who need to carefully allot their time.

Even though nurses acknowledged their appreciation for both nursing roles, tensions existed around finding time to complete all of their work. PHNs experienced frustrations with the imbalance of time allotted and time required to effectively manage their role in the NFP program. Rural PHNs discussed times where they were unable to finish nursing tasks within their workday and would miss breaks, skip lunch, or work late to complete them. PHNs described feelings of frustration related to their lack of time and inability to successfully balance both roles as emphasized by this nurse:

I feel that the amount of work that I have, because I do public health nursing as well as NFP work, that I'm finding the amount of time NFP work is taking is higher than the amount of time I have allocated for NFP, and so I feel that that is an imbalance and it's really hard to logistically sort of keep up to date with my generalist role and all the other things I do as a public health nurse and the NFP work at the same time. (PHN 10)

The quote above describes the time associated with the NFP program and the logistics of the generalist role, reflecting a desire to provide competent care while highlighting her need for more time and was repeated through many interviews. This imbalance of time was discouraging for nurses who wanted to perform well in all roles while they became more proficient within the NFP role.

### Staying connected

PHNs visiting clients and meeting face-to-face, preferably in their home environments, is fundamental to the NFP program and requires regular communication between nurse and client, nurses on the NFP team, and nurse and NFP supervisor. Communication is particularly important given the complex nature of clients and the specificity of the NFP intervention. NFP PHNs often communicate with clients to confirm appointments and follow up with nursing care in between home visits. Connecting with other NFP nurses and their supervisors helped PHNs to feel supported in their nursing practice, contemplate difficult situations, and inform clinical actions. However, concerns with the ability to freely communicate as a rural PHN in NFP were noted.

Communication difficulties appeared to cause barriers to visiting the client in her home environment. Not being able to reach rural clients interfered with the ability to provide nursing care and had a negative impact on practice as described in this participant quote, “A lot of our clients don’t have ongoing telephone. Like, you just can’t always get a hold of them. Their phones don’t have minutes [unable to make or receive telephone calls] on them or have been disconnected” (PHN 9). Nurses shared communication strategies to connect with clients, including knocking on clients’ apartment windows, contacting a family member, texting, or emailing. Overall, the most difficult communication barrier was when clients lacked a working cell phone and it made simple tasks (e.g. scheduling appointment, reminders, etc.) more complex and laborious:

Within a drug life of owing money to people, the simple things like trying to contact them [NFP clients] on a phone to make a next appointment, well they just pawned their phone off in order to pay for the immediate concerns. (PHN 2)

PHNs had empathy for their NFP clients, understanding that they experienced frequent financial crises and rarely had consistent means of communication. However, the inability to easily connect with rural clients interfered with the delivery of NFP required elements, such as home visits and field supervision.

PHNs and supervisors navigated the lack of connection and communication associated with being the lone NFP nurse in their jurisdiction by devising innovative strategies to support NFP practices. Rural geography influenced regularly scheduled NFP team meetings and education, as suggested in this quote from a PHN, “Well one of the challenges for rural and remote is not being able to come in all the time for face-to-face education. I think that that can be more challenging” (PHN 4). Face-to-face connection was also valued by supervisors, “Getting to hear others’ experiences was probably one of the more valuable things. Actually having a face-to-face for the core education and hearing other nurses’ concerns, talking about ours, and sharing what’s worked was really valuable” (SUP 9). Some attempts from supervisors to support rural NFP nurses involved having rural nurses meet independently with each other, connect via videoconferencing, to spend a few hours with each rural nurse, and to advocate for more travel funding so urban teams could meet the rural nurse. Addressing the lack of connection and the needs of nurses practicing as the sole NFP nurse at their office location was an important consideration for supporting rural nursing.

Supportive supervisors who understood the demands and barriers associated with rural nursing were essential to the success of developing innovative solutions to support NFP delivery in rural communities. One supervisor (SUP 2) described the need for “teleconference etiquette” for urban nurses who often forgot about the rural nurse not physically present but attending meetings virtually:

The remote nurses and the rural nurses are pretty used to teleconferencing. But the team that meets in-person [in the city], they weren't as used to it. So, that took some time for them to get the etiquette around teleconferencing. For getting that whole team building, you need to be able to teleconference well. That was definitely challenging. But that has come over time and setting down some ground rules for etiquette.

The development of these group rules helped support the inclusion of the rural nurses on the NFP team, who were often not well known to the urban NFP nurses. Navigating lack of connection through the use of supportive strategies to facilitate effective communication between the NFP team helped to maintain program delivery despite geographical distance.

### Remaining flexible

Beyond balancing the demands of their nursing roles, PHNs working part-time in an NFP capacity struggled to remain flexible and client-centred to meet client needs in a timely manner. One nurse reflected on the difficulty of having a limited visiting schedule when working with busy clients, “Well just balancing work. If they [clients] do get a job, trying to work around fitting in seeing me. The fact that I only work part-time visiting, trying to get the time in within my working day” (PHN 8). Working in a part-time capacity further compounded the need to be flexible given the time limitations.

NFP nurses remained flexible to meet the challenge of their clients’ busy schedules, given the nurses’ limited availability for home visiting due to their dual role or part-time position in a rural office. At times this required rural nurses to become creative with their approaches to finding mutually agreeable times for home visits. Nurses attempted to visit primarily in home but remained flexible in meeting clients where they were at to optimize the time of both rural nurse and client, as evidenced in this quote:

Ideally, we want to be meeting in the home but if that's the deal breaker then meet where they're going to want to meet. Right? Whether it's a coffee shop or a park or whatever. So, I do have to be flexible. (PHN 3)

Remaining flexible to the meeting space, even though program requirements recommend primarily meeting in the home, helped rural nurses to provide NFP services to their clients.

In addition to the client-focused elements of the NFP program, rural nurses and their supervisors acquired flexible approaches to meet the supervisory components of NFP. Supervisors provided support to PHNs by participating in a variety of role-specific activities, including weekly reflection, team meetings, case conferences, and field supervision. On an annual basis, it is recommended that each PHN and NFP supervisor dyad participate in at least three joint home visits with an NFP client. Scheduling and completing these joint home visits was another concern exacerbated by rural geography. NFP clients living in rural communities were often unpredictable and clients could cancel without notice. If a supervisor was traveling from an urban centre, there was limited means to connect prior to the scheduled visit as compared to nurses working within the same physical space as their supervisor. Most supervisors traveled extensive distances to reach rural nurses so cancelled home visits resulted in additional lost time and resources. Some nurses perceived this to be a significant challenge as suggested in the following quote:

It's harder for my team leader and I to get together for joint home visits or if she makes the trip all the way over here… and I have a couple of visits but for that day, you know, people might change or cancel or rebook or not show, and so it's harder versus if we were in the same office I'd be able to quickly say even within 15 minutes, I'm heading out to this home visit, everything's a go, do you want to come with me? So, the joint home visits are more of a challenge to be able to book and get in for the fidelity requirements for NFP. (PHN 4)

This participant quote also reflects the respect that the rural nurse had for the supervisor’s travel time and the need to efficiently manage time. Across all narratives, PHNs and supervisors were flexible, considerate, and committed to finding meaningful methods of responding to client needs, while meeting the requirements of the NFP program. Solution-focused, flexible approaches allowed the required elements of the NFP program to be delivered despite the constraints placed upon nurses and supervisors because of rural geography.

Being part of the NFP program helped clients to address short and long-term goals. At times, remaining flexible meant that immediate crises were dealt with regardless of the planned NFP activity. For example, clients’ needs for food or safety were prioritized. Supervisors noticed the flexibility required when providing nursing care in the NFP program as indicated in the following quote:

I know one of the nurses before a joint visit I did with her just last week, she had talked about the client we were going to see and walked through how she'd like to approach that specific topic and area of concern. And then when we were there together, she went through her plan [with the mother] and then just [adjusted it]. It was really great to be able to see her carry out that plan and be flexible and change it up as she needed to (SUP 1).

Despite the gravity of any presenting problem, NFP nurses directed clients to focus on solutions and continue working towards meeting program goals and future achievements.

### Providing complex and essential services

Overall, rural nurses noted that the combination of working with clients who were experiencing complex challenges in their lives and living in environments with limited available services meant that NFP was a vital and essential resource. Nurses reflected on the circumstances of clients, which revealed a wide array of health and social concerns, as is common with clients enrolled in NFP. The multiple, chronic, and complex nature of her client’s life and situation was shared by this PHN:

[The client has an] IQ of about 70 or 75 and she was born super premature, she was 26 weeks I think, and had all kinds of physical issues when she was an infant. She was in 33 foster homes by the time she was 15 and has some severe attachment issues. It was really sad. But anyway, her baby was apprehended ... But just complicated, holy cow. (PHN 5)

Other client concerns and health challenges mentioned by participants included: mental health disorders, substance use and addictions, violence, child apprehension, attachment disorders, extreme poverty, family members (parents/grandparents) with health and social problems, intellectual and learning disabilities, young maternal age, and infants with health concerns. This is not an exhaustive list but begins to outline the diverse and difficult situations experienced by clients in the NFP program and occurs in rural communities where other services are limited.

Nurses noted the importance of NFP in the lives of their rural clients, especially in the face of extreme financial and social disparity. NFP nurses working in rural communities were keenly aware of the lack of services available for their clients. Limited availability of health and social care providers, specialist services, accessible transit, difficulty reaching food banks, and other social services were mentioned as barriers to clients’ well-being. For these reasons, rural clients were greatly in need of, and receptive to, the NFP program:

It’s a small, rural town so people don't have access to huge amounts [of services]. I mean in [the city] there are programs about every single thing possible. There's much less to offer here. I think when people are given the opportunity to do a program like this they want to do it because there's so little else in the town. (PHN 8)

Supervisors also acknowledged the limited available services but recognised the ease and connection between service providers, “You have a smaller number of service providers and they already know each other often anyway” (SUP 6). Given the limited rural resources available and accessible, along with the complexities of clients in the NFP program, NFP was perceived by participants as an essential service within their rural communities.

## Discussion

The narrative derived from this analysis reflects both structural (weathering realities) and adaptive (being the sole NFP nurse, guarding time, staying connected, and remaining flexible) themes, and outlines the experience of providing a complex and essential service in rural British Columbia communities. The factors and challenges of providing a nursing intervention in rural settings, within the context of home-visiting with economically and socially disadvantaged young mothers, were presented. While the disadvantage experienced by NFP clients in rural communities is not exclusive to their geography [42], participants’ descriptions of their clients illustrated the complexity of existing situations within rural settings. PHNs and NFP supervisors are well-positioned to identify the modifications that are required to support the delivery of NFP in rural communities.

Because of the challenges associated with delivering nursing care to clients in rural settings, it is important to be able to identify rural communities where these services are required. NFP nurses need to articulate what classifies as rural in order to effectively determine how to best provide services to these populations. Definitions of rural included geographical and cultural considerations and a population size that is consistent with that used by Statistics Canada [41]. Nurses characterized rural similarly to the United States Census Bureau: that which is not urban is rural [43]. More importantly, participants’ recognition of a rural-urban continuum rather than a dichotomous, fixed definition is frequently used in research, supported in rural literature, and may be useful in determining rural health priorities, outcomes, or initiatives [6, 38, 44–46]. The use of a continuum is inclusive of, and sensitive to, the complexities associated with health geography including concepts such as rural-urban interface, ruralized urban, old rural, and new rural [45, 47–49]. The use of a rural-urban continuum will aid decision makers and rural practitioners in identifying what resonates as rural for their communities when implementing and delivering public health services. This is important so that all geographical areas are considered when delivering NFP, or any health intervention, rather than focusing specifically on urban or farmlands or remote communities; instead, a broad definition allows for consideration of small towns, country areas, or any area that doesn’t fall into a dichotomized definition.

In this analysis, time was valued and guarded as a resource constrained by rural practice and efficient communication was considered a cornerstone of effective delivery of NFP in rural communities. Challenges associated with rural practice included the place-based realities of extreme weather conditions, traveling far distances, and limited access to community services for clients and have been previously reported as rural practice issues [50–53]. These findings stress the importance of NFP in rural communities that often lack health and social services for this population yet still have clients with complex health and social concerns. Because rural communities lack many health and social services [4, 6, 54, 55], enhanced PHN services are vital for reaching vulnerable populations of girls and young women and their infants. Previous research has identified that rural health care providers have been neglected in research and there is a void of policies that address extreme weather for rural practitioners [53]. The implications arising from this analysis also call for health research and policy that support the realities of nurses working in rural communities.

Although rural-practicing nurses accept the nature of their geography as a normal occurrence, these environmental conditions must be considered when offering NFP in rural communities. Extreme weather and relative difficulty accessing roadways in some areas are issues that require some examination for senior decision makers. Existing studies have also reported complexities associated with rural public health nursing, including weathering difficult geographical terrain, travelling extensive distances, and working with limited available services [13, 15].

Addressing issues of isolation for PHNs who are not co-located in the same office as other NFP peers or their NFP supervisor could help support decision-making and offer opportunities for peer debriefing that may more naturally occur in urban centres. The use of videoconferencing opportunities could address, at least in part, some of the issues of isolation faced by rural NFP nurses. Where possible nurses may be encouraged to attend face-to-face meetings. However, when teleconferencing must occur, supervisors could focus their attention to ensuring that group norms are such that they include the rural nurses not attending in-person.

Another study found that although rural nurses working as the lone PHN in an office enjoyed their autonomy and close community ties, they also felt disconnected from the main nursing offices [12]. Using technology to create connections for rural nurses could both enhance rural nursing practice as it relates to NFP and respond to fiscal restrictions. The development of clear meeting guidelines when rural nurses join in-person meetings via teleconferencing/videoconferencing may help rural nurses’ engagement and inclusion in group activities. Being able to actively participate in meetings via telephone or video will ensure all NFP nurses can discuss their work within a team setting, and to receive the support and guidance of their colleagues.

Rural NFP PHNs recognized that they are often required to travel long distances and were more concerned with time than distance. This may be because adequate time is necessary for providing good care to NFP clients. Previous research has noted the importance of rural supervisors in order to deal with demands of rural nursing practice including isolation, limited connection to other professionals, and opportunities to debrief with peers [18, 56, 57] and are consistent with the findings of this analysis. In addition, this study adds the advocacy role of supervisors to ensure that rural nurses have sufficient time for nursing practice. Using secure video-conferencing to provide nurse supervision during home-visiting with families may be one method that could address distance as a barrier to field supervision. Technology could be used to facilitate program supervision requirements and address issues of distance, time, and the unpredictable nature of home-visiting.

The need to guard time was particularly important for nurses who had dual roles at their health authority, practicing both as a generalist and an NFP nurse. Rural nursing practice can be challenging due to the multi-faceted skill set and nursing tasks required of the NFP nurse working in a dual role. Although not preferable, NFP nurses may be required to hold a dual role due to limited available staff, smaller populations, and the required cross-training of rural nurses, as reflected in previous literature [17]. Supervisors who are supportive and aware of rural nursing practice issues are well-situated to assist part-time or dual-role NFP PHNs in scheduling their time and advocating for their needs.

NFP may enhance nursing practice by addressing some long-standing concerns and issues constraining rural nurses. Multiple studies have found that rural nurses lack the necessary mentoring to feel competent in their nursing practice [18, 58, 59]. Other studies have considered the lack of consistent and regular educational opportunities as a barrier to rural nursing practice [58, 59]. Because the NFP program is structured to provide regular education, supervision, and team support, rural nurses have access to consistent interactions that could bolster their rural nursing practice and add to the contextual knowledge they already have about their rural communities.

### Strengths and limitations

This study focused on the experiences of PHNs and supervisors providing the NFP program to select rural communities in British Columbia, Canada and has both strengths and limitations. First, this study only captured one group of nurses at a specific point in the early stages of implementing the NFP program and did not consider the experiences of urban-situated nurses who also delivered NFP to some rural communities. This was not a comparison study, so it is unknown how geography affected nurses practicing in urban and suburban areas. Future evaluations will incorporate their experiences to broaden what is known about delivery of NFP in rural communities. Also, the experiences of rural clients were not considered; however, the focus of this analysis was specific to nursing practice and so the voices of nurses were fundamental in understanding how NFP is delivered in rural communities in British Columbia, Canada.

Strengths of the study include using a qualitative design that is specific to applied health sciences and allowed the nurses’ and supervisors’ experiences, through interviews, analysis, and interpretation, to guide the development of clinically-relevant implications for rural nurses, supervisors, and health policy decision makers. Additionally, because this study is embedded within a large process evaluation, there are opportunities for team discussion and exploration of rural issues, as well as longer-term follow up with the participants that will help to strengthen our growing understanding of nurses’ and supervisors’ experiences delivering NFP in rural British Columbia and Canadian communities with future analyses.

## Conclusions

This study contributes to the larger BCHCP process evaluation by being the first paper to explore the practices of PHNs delivering the NFP program in rural British Columbia. This research will help inform the modifications and adaptations required to the theory and intervention components of the NFP program within a rural context, for successful implementation and delivery in Canada, if the NFP is shown to be effective. The findings of this study constitute the basis for the development of a rural NFP model by providing an initial understanding of NFP nurse and supervisor experiences and identifies the program’s limitations within the rural British Columbia geographical context. On an international scale, study conclusions may provide guidance to other countries implementing NFP in similar geographic areas.

Exploring the experiences of NFP PHNs and their supervisors is necessary to determine how NFP can be successfully delivered in rural communities. Gaining a greater understanding of how NFP can support rural communities is vitally important given the strong evidentiary base behind NFP as an effective strategy to improve the health and well-being of socially and economically disadvantaged young girls and women, combined with the health disparities associated with rural-living. Future research will examine the experiences of all NFP nurses providing care to rural-dwelling clients to help inform the development of strategies that build success in program delivery. Because supervision was critical to rural NFP delivery, future research focusing on supervisors’ perspectives of meeting program supervision elements will enhance the development of a rural NFP model. Finally, the perspectives of senior decision-makers who guide organizational policies and funding models for NFP will be incorporated in the development of a Canadian model for rural NFP delivery, if the program is effective within a Canadian context.

## Additional file

Additional file 1: Interview Guide. Supplementary file 1 is the interview guide used during the interview process, specific to geography as a contextual influencing factor. (DOCX 13 kb)

## Abbreviations

BCHCP: British Columbia Healthy Connections Project
NFP: Nurse-Family Partnership
PHN(s): Public health nurse(s)
RCT: Randomized controlled trial
SUP: Supervisor
