# Supplementary material for: Weathering the rural reality: delivery of the Nurse-Family Partnership home visitation program in rural British Columbia, Canada
Source: BMC Nurs. 2019 May 2;18:17. doi: 10.1186/s12912-019-0341-3 (PMC6498595; doi:10.1186/s12912-019-0341-3)
Supplement: Supplementary file 1 — Interview Guide. Supplementary file 1 is the interview guide used during the interview process, specific to geography as a contextual influencing factor. (DOCX 13 kb) [file 12912_2019_341_MOESM1_ESM.docx]

**Interview Guide**

Hello, my name is (name) and I am the (position) on the BC Healthy Connections Project (BCHCP) Process Evaluation Research Team. As part of this study, you have given consent for me to interview you approximately every six months about your experiences with the Nurse-Family Partnership program as it is being delivered within the (BCHCP). You completed the first interview on (date of first interview) and today I would like to continue our conversation about your experiences with the Nurse-Family Partnership program. The interview today will last approximately 60 minutes.

Again, the overall purpose of this component of the scientific evaluation is to understand how the Nurse-Family Partnership (NFP) intervention is implemented and integrated into public health nursing practice and what changes occur over time. We are most interested in learning about your personal experiences working in the program, including the successes and challenges you have encountered throughout this process. There are no right or wrong answers.

Your participation is completely voluntary and we can stop the interview at anytime. You may also choose not to answer any questions that you do not feel comfortable answering. The information you share about your work in the NFP program will also remain confidential and will not be shared with anyone on your team or your supervisor. The data from all of the interviews will be synthesized and general broad themes will be summarized and shared back to all of the NFP teams and BCHCP stakeholders.

There are many different external factors that will influence the successful uptake of a new intervention. These factors may be related to characteristics of the nurse, the client, the Health Authority or the NFP intervention itself. Broader social, cultural, geographical or political factors can also influence the process of how the NFP is introduced and delivered in each Health Authority.

1.1 Describe the physical geography of the community from where you deliver the NFP and some of the attributes of the client groups you commonly work with.

a. How does the geography of your community influence your ability to deliver the NFP?

b. What challenges do you experience in maintaining fidelity to the model?

c. What innovative practice strategies have been developed within your team to understand and respond to these influences?

1.2 Describe the organizational context in which you deliver the NFP.

a. What organizational attributes have facilitated your ability to implement the NFP? Why?

b. What organizational attributes have made it challenging for you to deliver the NFP? Why?

As we wrap our conversation today, are there any additional comments that you would like to share with the research team about issues that influence how the NFP is being delivered within your Health Authority?
